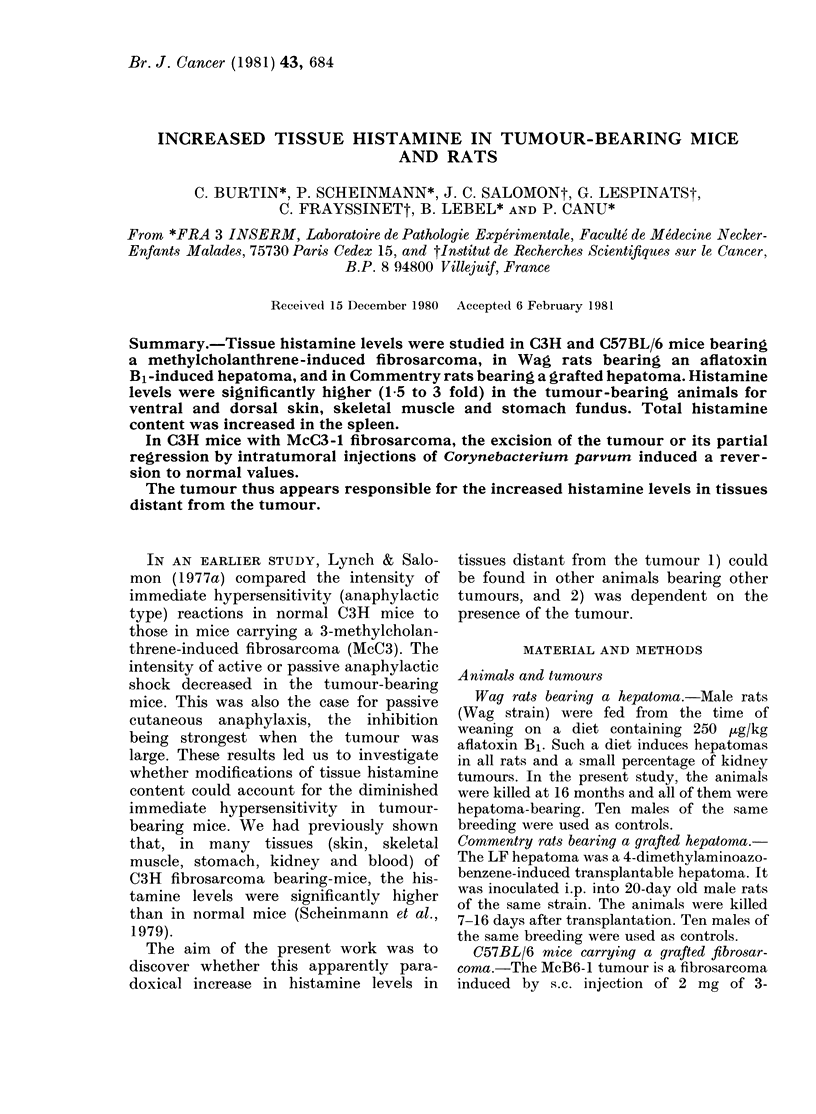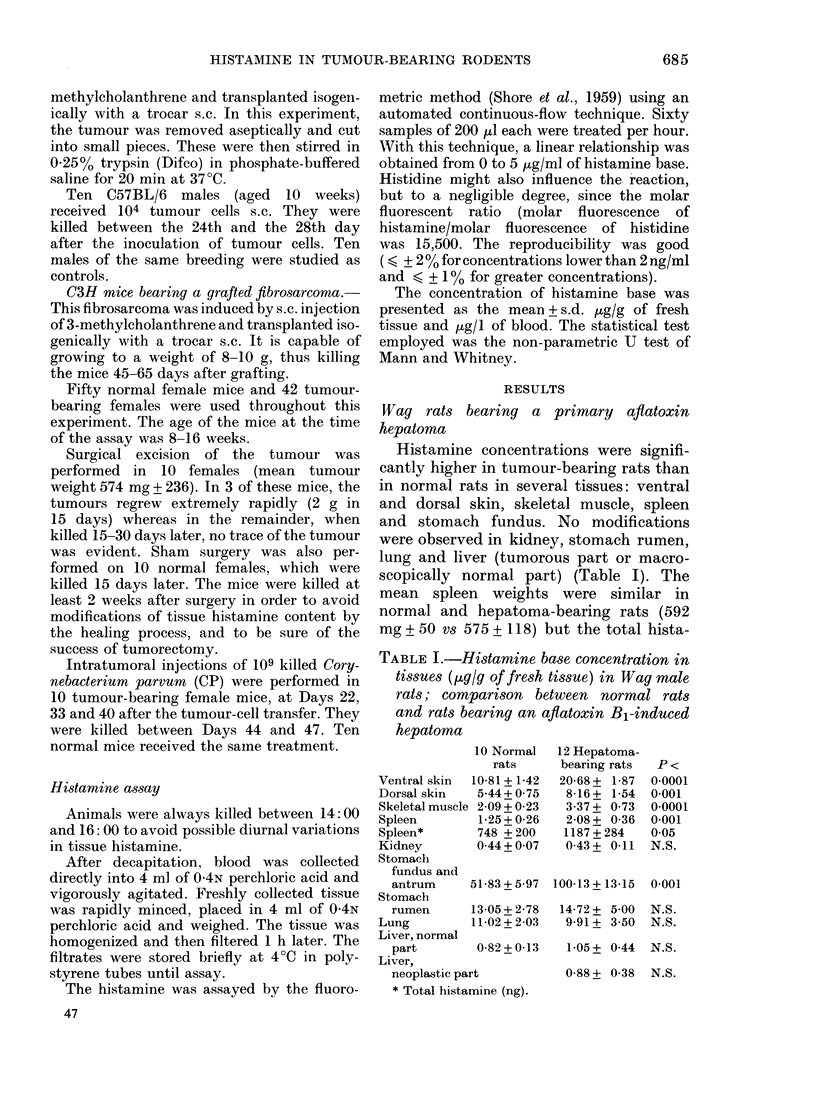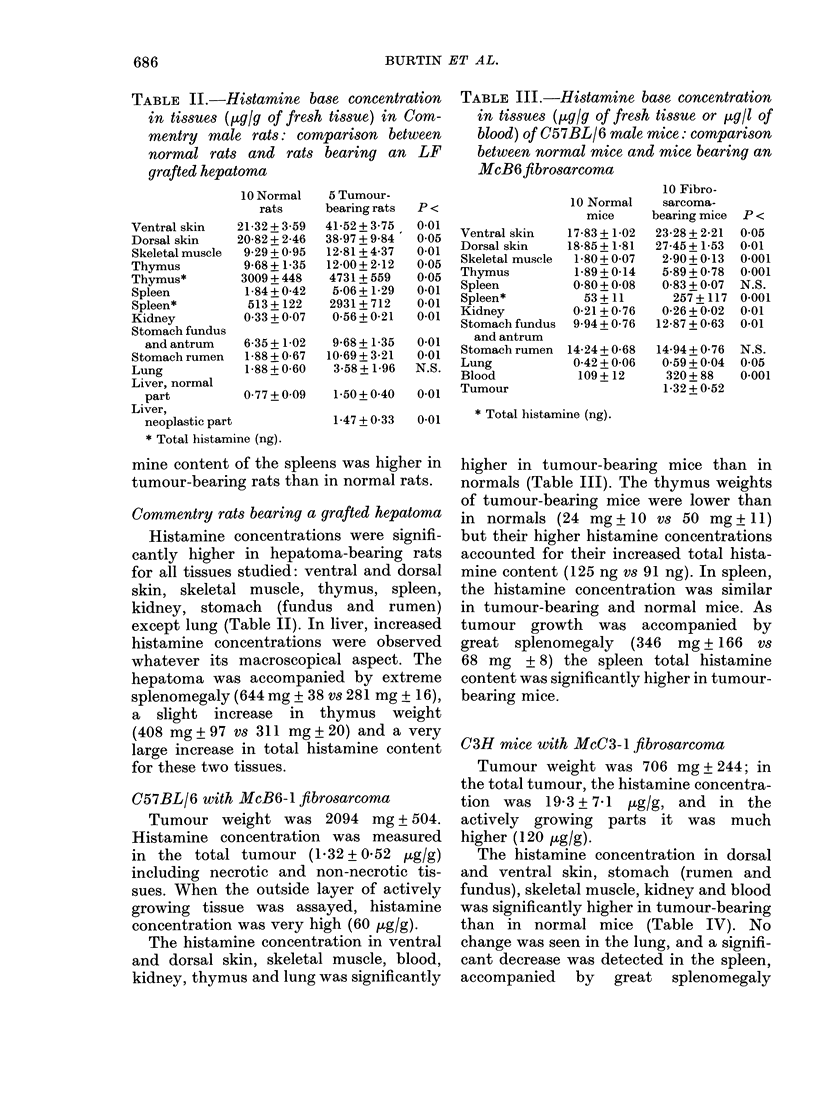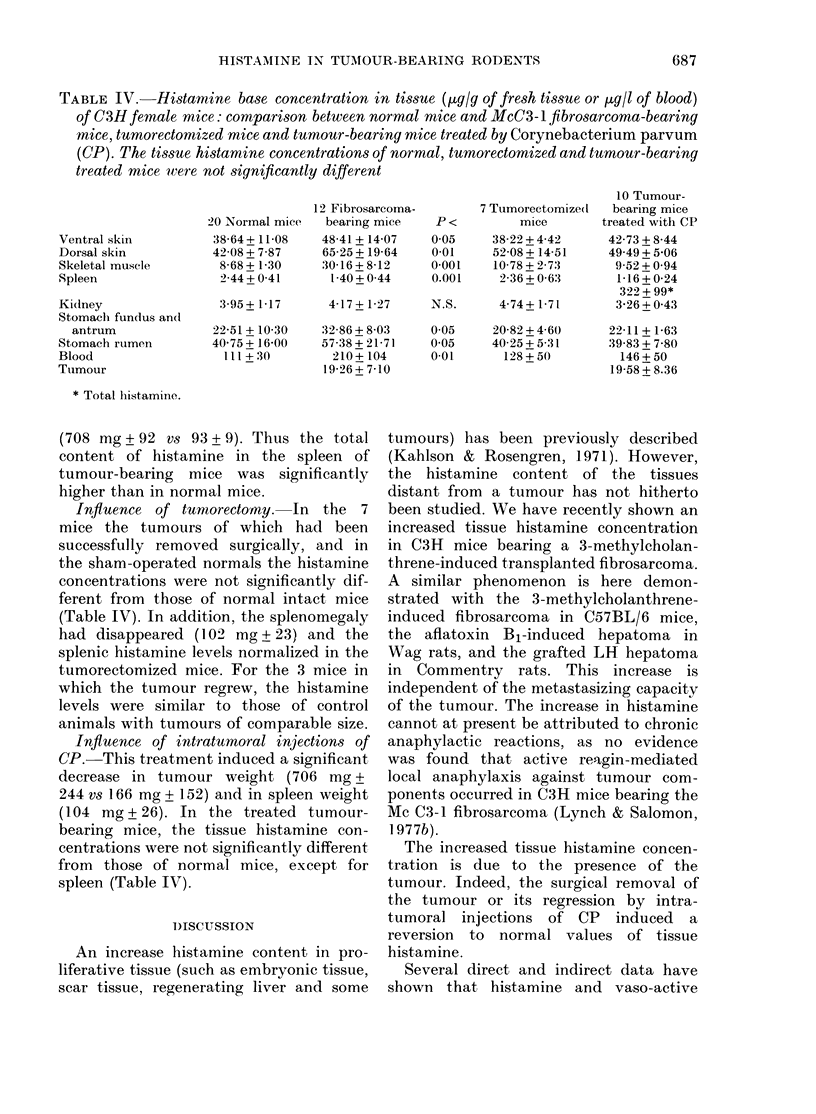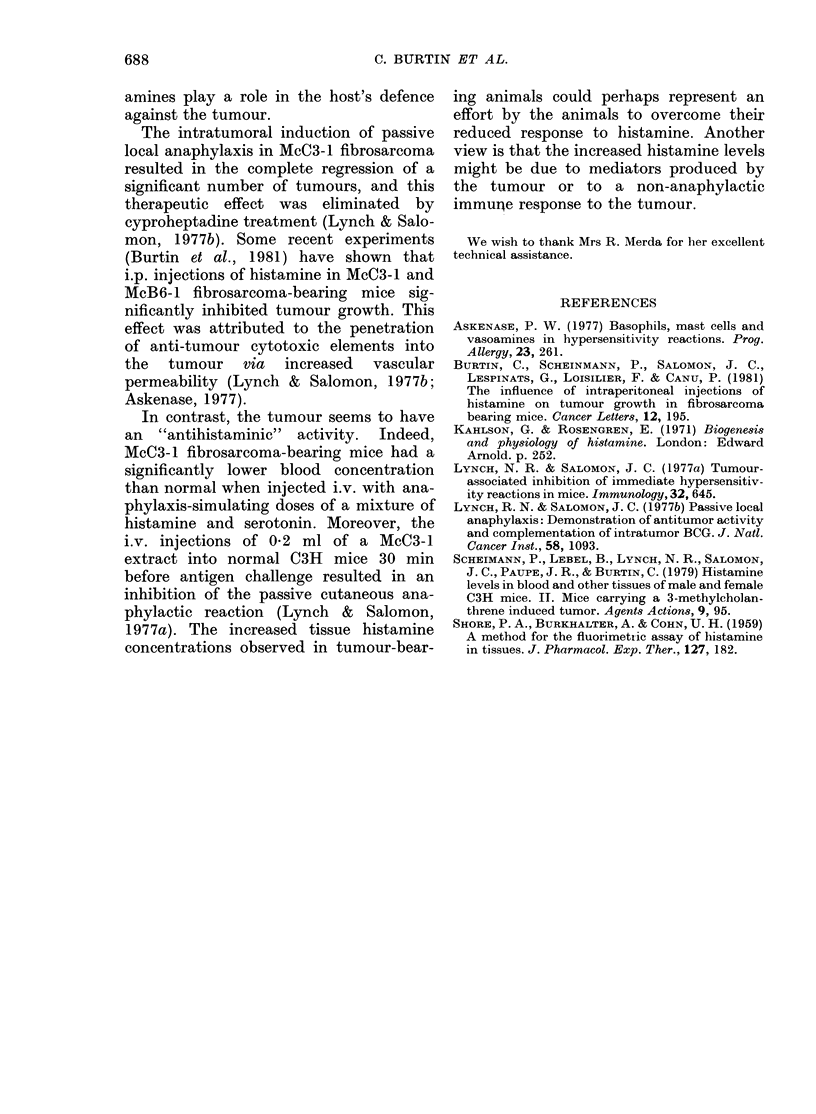# Increased tissue histamine in tumour-bearing mice and rats.

**DOI:** 10.1038/bjc.1981.99

**Published:** 1981-05

**Authors:** C. Burtin, P. Scheinmann, J. C. Salomon, G. Lespinats, C. Frayssinet, B. Lebel, P. Canu

## Abstract

Tissue histamine levels were studied in C3H and C57BL/6 mice bearing a methylcholanthrene-induced fibrosarcoma, in Wag rats bearing an aflatoxin B1-induced hepatoma, and in Commentry rats bearing a grafted hepatoma. Histamine levels were significantly higher (1.5 to 3 fold) in the tumour-bearing animals for ventral and dorsal skin, skeletal muscle and stomach fundus. Total histamine content was increased in the spleen. In C3H mice with McC3-1 fibrosarcoma, the excision of the tumour or its partial regression by intratumoral injections of corynebacterium parvum induced a reversion to normal values. The tumour thus appears responsible for the increased histamine levels in tissues distant from the tumour.


Br. J. Cancer (1981) **43**, 684

### INCREASED TISSUE HISTAMINE IN TUMOUR-BEARING MICE AND RATS

C. BURTIN*, P. SCHEINMANN*, J. C. SALOMON†, G. LESPINATS†, C. FRAYSSINET†, B. LEBEL* AND P. CANU*

*From *FRA 3 INSERM, Laboratoire de Pathologie Expérimentale, Faculté de Médecine Necker-Enfants Malades, 75730 Paris Cedex 15, and †Institut de Recherches Scientifiques sur le Cancer, B.P. 8 94800 Villejuif, France*

Received 15 December 1980 Accepted 6 February 1981

**Summary.—Tissue histamine levels were studied in C3H and C57BL/6 mice bearing a methylcholanthrene-induced fibrosarcoma, in Wag rats bearing an aflatoxin $B_1$-induced hepatoma, and in Commentry rats bearing a grafted hepatoma. Histamine levels were significantly higher (1·5 to 3 fold) in the tumour-bearing animals for ventral and dorsal skin, skeletal muscle and stomach fundus. Total histamine content was increased in the spleen.**

**In C3H mice with McC3-1 fibrosarcoma, the excision of the tumour or its partial regression by intratumoral injections of *Corynebacterium parvum* induced a reversion to normal values.**

**The tumour thus appears responsible for the increased histamine levels in tissues distant from the tumour.**

IN AN EARLIER STUDY, Lynch & Salomon (1977*a*) compared the intensity of immediate hypersensitivity (anaphylactic type) reactions in normal C3H mice to those in mice carrying a 3-methylcholanthrene-induced fibrosarcoma (McC3). The intensity of active or passive anaphylactic shock decreased in the tumour-bearing mice. This was also the case for passive cutaneous anaphylaxis, the inhibition being strongest when the tumour was large. These results led us to investigate whether modifications of tissue histamine content could account for the diminished immediate hypersensitivity in tumour-bearing mice. We had previously shown that, in many tissues (skin, skeletal muscle, stomach, kidney and blood) of C3H fibrosarcoma bearing-mice, the histamine levels were significantly higher than in normal mice (Scheinmann *et al.*, 1979).

The aim of the present work was to discover whether this apparently paradoxical increase in histamine levels in tissues distant from the tumour 1) could be found in other animals bearing other tumours, and 2) was dependent on the presence of the tumour.

### MATERIAL AND METHODS

### *Animals and tumours*

*Wag rats bearing a hepatoma.*—Male rats (Wag strain) were fed from the time of weaning on a diet containing 250 μg/kg aflatoxin $B_1$. Such a diet induces hepatomas in all rats and a small percentage of kidney tumours. In the present study, the animals were killed at 16 months and all of them were hepatoma-bearing. Ten males of the same breeding were used as controls.

*Commentry rats bearing a grafted hepatoma.*—The LF hepatoma was a 4-dimethylaminoazobenzene-induced transplantable hepatoma. It was inoculated i.p. into 20-day old male rats of the same strain. The animals were killed 7–16 days after transplantation. Ten males of the same breeding were used as controls.

*C57BL/6 mice carrying a grafted fibrosarcoma.*—The McB6-1 tumour is a fibrosarcoma induced by s.c. injection of 2 mg of 3-

methylcholanthrene and transplanted isogenically with a trocar s.c. In this experiment, the tumour was removed aseptically and cut into small pieces. These were then stirred in 0·25% trypsin (Difco) in phosphate-buffered saline for 20 min at 37 °C.

Ten C57BL/6 males (aged 10 weeks) received $10^4$ tumour cells s.c. They were killed between the 24th and the 28th day after the inoculation of tumour cells. Ten males of the same breeding were studied as controls.

*C3H mice bearing a grafted fibrosarcoma.*—This fibrosarcoma was induced by s.c. injection of 3-methylcholanthrene and transplanted isogenically with a trocar s.c. It is capable of growing to a weight of 8–10 g, thus killing the mice 45–65 days after grafting.

Fifty normal female mice and 42 tumour-bearing females were used throughout this experiment. The age of the mice at the time of the assay was 8–16 weeks.

Surgical excision of the tumour was performed in 10 females (mean tumour weight 574 mg ± 236). In 3 of these mice, the tumours regrew extremely rapidly (2 g in 15 days) whereas in the remainder, when killed 15–30 days later, no trace of the tumour was evident. Sham surgery was also performed on 10 normal females, which were killed 15 days later. The mice were killed at least 2 weeks after surgery in order to avoid modifications of tissue histamine content by the healing process, and to be sure of the success of tumorectomy.

Intratumoral injections of $10^9$ killed *Corynebacterium parvum* (CP) were performed in 10 tumour-bearing female mice, at Days 22, 33 and 40 after the tumour-cell transfer. They were killed between Days 44 and 47. Ten normal mice received the same treatment.

### *Histamine assay*

Animals were always killed between 14:00 and 16:00 to avoid possible diurnal variations in tissue histamine.

After decapitation, blood was collected directly into 4 ml of 0·4N perchloric acid and vigorously agitated. Freshly collected tissue was rapidly minced, placed in 4 ml of 0·4N perchloric acid and weighed. The tissue was homogenized and then filtered 1 h later. The filtrates were stored briefly at 4°C in polystyrene tubes until assay.

The histamine was assayed by the fluorometric method (Shore *et al.*, 1959) using an automated continuous-flow technique. Sixty samples of 200 μl each were treated per hour. With this technique, a linear relationship was obtained from 0 to 5 μg/ml of histamine base. Histidine might also influence the reaction, but to a negligible degree, since the molar fluorescent ratio (molar fluorescence of histamine/molar fluorescence of histidine was 15,500. The reproducibility was good ($\leqslant \pm 2\%$ for concentrations lower than 2 ng/ml and $\leqslant \pm 1\%$ for greater concentrations).

The concentration of histamine base was presented as the mean ± s.d. μg/g of fresh tissue and μg/l of blood. The statistical test employed was the non-parametric U test of Mann and Whitney.

### RESULTS

### *Wag rats bearing a primary aflatoxin hepatoma*

Histamine concentrations were significantly higher in tumour-bearing rats than in normal rats in several tissues: ventral and dorsal skin, skeletal muscle, spleen and stomach fundus. No modifications were observed in kidney, stomach rumen, lung and liver (tumorous part or macroscopically normal part) (Table I). The mean spleen weights were similar in normal and hepatoma-bearing rats (592 mg ± 50 *vs* 575 ± 118) but the total hista-

TABLE I.—*Histamine base concentration in tissues (μg/g of fresh tissue) in Wag male rats; comparison between normal rats and rats bearing an aflatoxin $B_1$-induced hepatoma*

| | 10 Normal rats | 12 Hepatoma-bearing rats | $P <$ |
|---|---|---|---|
| Ventral skin | 10·81 ± 1·42 | 20·68 ± 1·87 | 0·0001 |
| Dorsal skin | 5·44 ± 0·75 | 8·16 ± 1·54 | 0·001 |
| Skeletal muscle | 2·09 ± 0·23 | 3·37 ± 0·73 | 0·0001 |
| Spleen | 1·25 ± 0·26 | 2·08 ± 0·36 | 0·001 |
| Spleen* | 748 ± 200 | 1187 ± 284 | 0·05 |
| Kidney | 0·44 ± 0·07 | 0·43 ± 0·11 | N.S. |
| Stomach fundus and antrum | 51·83 ± 5·97 | 100·13 ± 13·15 | 0·001 |
| Stomach rumen | 13·05 ± 2·78 | 14·72 ± 5·00 | N.S. |
| Lung | 11·02 ± 2·03 | 9·91 ± 3·50 | N.S. |
| Liver, normal part | 0·82 ± 0·13 | 1·05 ± 0·44 | N.S. |
| Liver, neoplastic part | | 0·88 ± 0·38 | N.S. |

* Total histamine (ng).

TABLE II.—*Histamine base concentration in tissues (μg/g of fresh tissue) in Commentry male rats: comparison between normal rats and rats bearing an LF grafted hepatoma*

| | 10 Normal rats | 5 Tumour-bearing rats | $P <$ |
|---|---|---|---|
| Ventral skin | 21·32 ± 3·59 | 41·52 ± 3·75 | 0·01 |
| Dorsal skin | 20·82 ± 2·46 | 38·97 ± 9·84 | 0·05 |
| Skeletal muscle | 9·29 ± 0·95 | 12·81 ± 4·37 | 0·01 |
| Thymus | 9·68 ± 1·35 | 12·00 ± 2·12 | 0·05 |
| Thymus* | 3009 ± 448 | 4731 ± 559 | 0·05 |
| Spleen | 1·84 ± 0·42 | 5·06 ± 1·29 | 0·01 |
| Spleen* | 513 ± 122 | 2931 ± 712 | 0·01 |
| Kidney | 0·33 ± 0·07 | 0·56 ± 0·21 | 0·01 |
| Stomach fundus and antrum | 6·35 ± 1·02 | 9·68 ± 1·35 | 0·01 |
| Stomach rumen | 1·88 ± 0·67 | 10·69 ± 3·21 | 0·01 |
| Lung | 1·88 ± 0·60 | 3·58 ± 1·96 | N.S. |
| Liver, normal part | 0·77 ± 0·09 | 1·50 ± 0·40 | 0·01 |
| Liver, neoplastic part | | 1·47 ± 0·33 | 0·01 |

* Total histamine (ng).

TABLE III.—*Histamine base concentration in tissues (μg/g of fresh tissue or μg/l of blood) of C57BL/6 male mice: comparison between normal mice and mice bearing an McB6 fibrosarcoma*

| | 10 Normal mice | 10 Fibro-sarcoma-bearing mice | $P <$ |
|---|---|---|---|
| Ventral skin | 17·83 ± 1·02 | 23·28 ± 2·21 | 0·05 |
| Dorsal skin | 18·85 ± 1·81 | 27·45 ± 1·53 | 0·01 |
| Skeletal muscle | 1·80 ± 0·07 | 2·90 ± 0·13 | 0·001 |
| Thymus | 1·89 ± 0·14 | 5·89 ± 0·78 | 0·001 |
| Spleen | 0·80 ± 0·08 | 0·83 ± 0·07 | N.S. |
| Spleen* | 53 ± 11 | 257 ± 117 | 0·001 |
| Kidney | 0·21 ± 0·76 | 0·26 ± 0·02 | 0·01 |
| Stomach fundus and antrum | 9·94 ± 0·76 | 12·87 ± 0·63 | 0·01 |
| Stomach rumen | 14·24 ± 0·68 | 14·94 ± 0·76 | N.S. |
| Lung | 0·42 ± 0·06 | 0·59 ± 0·04 | 0·05 |
| Blood | 109 ± 12 | 320 ± 88 | 0·001 |
| Tumour | | 1·32 ± 0·52 | |

* Total histamine (ng).

mine content of the spleens was higher in tumour-bearing rats than in normal rats.

*Commentry rats bearing a grafted hepatoma*

Histamine concentrations were significantly higher in hepatoma-bearing rats for all tissues studied: ventral and dorsal skin, skeletal muscle, thymus, spleen, kidney, stomach (fundus and rumen) except lung (Table II). In liver, increased histamine concentrations were observed whatever its macroscopical aspect. The hepatoma was accompanied by extreme splenomegaly (644 mg ± 38 *vs* 281 mg ± 16), a slight increase in thymus weight (408 mg ± 97 *vs* 311 mg ± 20) and a very large increase in total histamine content for these two tissues.

*C57BL/6 with McB6-1 fibrosarcoma*

Tumour weight was 2094 mg ± 504. Histamine concentration was measured in the total tumour (1·32 ± 0·52 μg/g) including necrotic and non-necrotic tissues. When the outside layer of actively growing tissue was assayed, histamine concentration was very high (60 μg/g).

The histamine concentration in ventral and dorsal skin, skeletal muscle, blood, kidney, thymus and lung was significantly higher in tumour-bearing mice than in normals (Table III). The thymus weights of tumour-bearing mice were lower than in normals (24 mg ± 10 *vs* 50 mg ± 11) but their higher histamine concentrations accounted for their increased total histamine content (125 ng *vs* 91 ng). In spleen, the histamine concentration was similar in tumour-bearing and normal mice. As tumour growth was accompanied by great splenomegaly (346 mg ± 166 *vs* 68 mg ± 8) the spleen total histamine content was significantly higher in tumour-bearing mice.

*C3H mice with McC3-1 fibrosarcoma*

Tumour weight was 706 mg ± 244; in the total tumour, the histamine concentration was 19·3 ± 7·1 μg/g, and in the actively growing parts it was much higher (120 μg/g).

The histamine concentration in dorsal and ventral skin, stomach (rumen and fundus), skeletal muscle, kidney and blood was significantly higher in tumour-bearing than in normal mice (Table IV). No change was seen in the lung, and a significant decrease was detected in the spleen, accompanied by great splenomegaly

TABLE IV.—*Histamine base concentration in tissue (μg/g of fresh tissue or μg/l of blood) of C3H female mice: comparison between normal mice and McC3-1 fibrosarcoma-bearing mice, tumorectomized mice and tumour-bearing mice treated by* Corynebacterium parvum *(CP). The tissue histamine concentrations of normal, tumorectomized and tumour-bearing treated mice were not significantly different*

| | 20 Normal mice | 12 Fibrosarcoma-bearing mice | $P <$ | 7 Tumorectomized mice | 10 Tumour-bearing mice treated with CP |
|---|---|---|---|---|---|
| Ventral skin | 38·64 ± 11·08 | 48·41 ± 14·07 | 0·05 | 38·22 ± 4·42 | 42·73 ± 8·44 |
| Dorsal skin | 42·08 ± 7·87 | 65·25 ± 19·64 | 0·01 | 52·08 ± 14·51 | 49·49 ± 5·06 |
| Skeletal muscle | 8·68 ± 1·30 | 30·16 ± 8·12 | 0·001 | 10·78 ± 2·73 | 9·52 ± 0·94 |
| Spleen | 2·44 ± 0·41 | 1·40 ± 0·44 | 0.001 | 2·36 ± 0·63 | 1·16 ± 0·24<br>322 ± 99* |
| Kidney | 3·95 ± 1·17 | 4·17 ± 1·27 | N.S. | 4·74 ± 1·71 | 3·26 ± 0·43 |
| Stomach fundus and antrum | 22·51 ± 10·30 | 32·86 ± 8·03 | 0·05 | 20·82 ± 4·60 | 22·11 ± 1·63 |
| Stomach rumen | 40·75 ± 16·00 | 57·38 ± 21·71 | 0·05 | 40·25 ± 5·31 | 39·83 ± 7·80 |
| Blood | 111 ± 30 | 210 ± 104 | 0·01 | 128 ± 50 | 146 ± 50 |
| Tumour | | 19·26 ± 7·10 | | | 19·58 ± 8.36 |

* Total histamine.

(708 mg ± 92 *vs* 93 ± 9). Thus the total content of histamine in the spleen of tumour-bearing mice was significantly higher than in normal mice.

*Influence of tumorectomy.*—In the 7 mice the tumours of which had been successfully removed surgically, and in the sham-operated normals the histamine concentrations were not significantly different from those of normal intact mice (Table IV). In addition, the splenomegaly had disappeared (102 mg ± 23) and the splenic histamine levels normalized in the tumorectomized mice. For the 3 mice in which the tumour regrew, the histamine levels were similar to those of control animals with tumours of comparable size.

*Influence of intratumoral injections of CP.*—This treatment induced a significant decrease in tumour weight (706 mg ± 244 *vs* 166 mg ± 152) and in spleen weight (104 mg ± 26). In the treated tumour-bearing mice, the tissue histamine concentrations were not significantly different from those of normal mice, except for spleen (Table IV).

### DISCUSSION

An increase histamine content in proliferative tissue (such as embryonic tissue, scar tissue, regenerating liver and some tumours) has been previously described (Kahlson & Rosengren, 1971). However, the histamine content of the tissues distant from a tumour has not hitherto been studied. We have recently shown an increased tissue histamine concentration in C3H mice bearing a 3-methylcholanthrene-induced transplanted fibrosarcoma. A similar phenomenon is here demonstrated with the 3-methylcholanthrene-induced fibrosarcoma in C57BL/6 mice, the aflatoxin $B_1$-induced hepatoma in Wag rats, and the grafted LH hepatoma in Commentry rats. This increase is independent of the metastasizing capacity of the tumour. The increase in histamine cannot at present be attributed to chronic anaphylactic reactions, as no evidence was found that active reagin-mediated local anaphylaxis against tumour components occurred in C3H mice bearing the Mc C3-1 fibrosarcoma (Lynch & Salomon, 1977*b*).

The increased tissue histamine concentration is due to the presence of the tumour. Indeed, the surgical removal of the tumour or its regression by intratumoral injections of CP induced a reversion to normal values of tissue histamine.

Several direct and indirect data have shown that histamine and vaso-active amines play a role in the host's defence against the tumour.

The intratumoral induction of passive local anaphylaxis in McC3-1 fibrosarcoma resulted in the complete regression of a significant number of tumours, and this therapeutic effect was eliminated by cyproheptadine treatment (Lynch & Salomon, 1977*b*). Some recent experiments (Burtin *et al.*, 1981) have shown that i.p. injections of histamine in McC3-1 and McB6-1 fibrosarcoma-bearing mice significantly inhibited tumour growth. This effect was attributed to the penetration of anti-tumour cytotoxic elements into the tumour *via* increased vascular permeability (Lynch & Salomon, 1977*b*; Askenase, 1977).

In contrast, the tumour seems to have an "antihistaminic" activity. Indeed, McC3-1 fibrosarcoma-bearing mice had a significantly lower blood concentration than normal when injected i.v. with anaphylaxis-simulating doses of a mixture of histamine and serotonin. Moreover, the i.v. injections of 0·2 ml of a McC3-1 extract into normal C3H mice 30 min before antigen challenge resulted in an inhibition of the passive cutaneous anaphylactic reaction (Lynch & Salomon, 1977*a*). The increased tissue histamine concentrations observed in tumour-bearing animals could perhaps represent an effort by the animals to overcome their reduced response to histamine. Another view is that the increased histamine levels might be due to mediators produced by the tumour or to a non-anaphylactic immune response to the tumour.

We wish to thank Mrs R. Merda for her excellent technical assistance.

### REFERENCES

ASKENASE, P. W. (1977) Basophils, mast cells and vasoamines in hypersensitivity reactions. *Prog. Allergy*, **23**, 261.

BURTIN, C., SCHEINMANN, P., SALOMON, J. C., LESPINATS, G., LOISILIER, F. & CANU, P. (1981) The influence of intraperitoneal injections of histamine on tumour growth in fibrosarcoma bearing mice. *Cancer Letters*, **12**, 195.

KAHLSON, G. & ROSENGREN, E. (1971) *Biogenesis and physiology of histamine*. London: Edward Arnold. p. 252.

LYNCH, N. R. & SALOMON, J. C. (1977*a*) Tumour-associated inhibition of immediate hypersensitivity reactions in mice. *Immunology*, **32**, 645.

LYNCH, R. N. & SALOMON, J. C. (1977*b*) Passive local anaphylaxis: Demonstration of antitumor activity and complementation of intratumor BCG. *J. Natl. Cancer Inst.*, **58**, 1093.

SCHEIMANN, P., LEBEL, B., LYNCH, N. R., SALOMON, J. C., PAUPE, J. R., & BURTIN, C. (1979) Histamine levels in blood and other tissues of male and female C3H mice. II. Mice carrying a 3-methylcholanthrene induced tumor. *Agents Actions*, **9**, 95.

SHORE, P. A., BURKHALTER, A. & COHN, U. H. (1959) A method for the fluorimetric assay of histamine in tissues. *J. Pharmacol. Exp. Ther.*, **127**, 182.